# Pharmacological targeting of glucose-6-phosphate dehydrogenase in human erythrocytes by Bay 11–7082, parthenolide and dimethyl fumarate

**DOI:** 10.1038/srep28754

**Published:** 2016-06-29

**Authors:** Mehrdad Ghashghaeinia, Daniela Giustarini, Pavla Koralkova, Martin Köberle, Kousi Alzoubi, Rosi Bissinger, Zohreh Hosseinzadeh, Peter Dreischer, Ingolf Bernhardt, Florian Lang, Mahmoud Toulany, Thomas Wieder, Renata Mojzikova, Ranieri Rossi, Ulrich Mrowietz

**Affiliations:** 1 Psoriasis-Center, Department of Dermatology, University Medical Center Schleswig-Holstein, Campus Kiel, Schittenhelmstr. 7, Kiel, 24105, Germany; 2 Department of Life Sciences, Laboratory of Pharmacology and Toxicology, University of Siena, Via A Moro 2, 53100, Siena, Italy; 3 Department of Biology, Faculty of Medicine and Dentistry Palacky University, Hnevotinska 3, 77515 Olomouc, Czech Republic; 4 Department of Dermatology and Allergy, Biedersteinerstr. 29, Technische Universität München, 80802 München, Germany; 5 Department of Cardiology, Vascular Medicine and Physiology, University of Tübingen, Gmelinstr. 5, 72076, Tübingen, Germany; 6 Centre for Ophthalmology, Institute for Ophthalmic Research, Eberhard-Karls-University Tübingen, Frondsbergstr. 23, 72076 Tübingen, Germany; 7 Institute of Physiology II, Keplerstr. 15, Eberhard Karls University of Tübingen, 72074 Tübingen, Germany; 8 Laboratory of Biophysics, Saarland University, Campus A2.4, 66123 Saarbrücken, Germany; 9 Division of Radiobiology and Molecular Environmental Research, Department of Radiation Oncology, Roentgenweg 11, Eberhard Karls University Tübingen, 72076 Tübingen, Germany; 10 Department of Dermatology; Eberhard Karls University, Tübingen, Germany

## Abstract

In mature erythrocytes, glucose-6-phosphate dehydrogenase (G6PDH) and 6-phosphogluconate dehydrogenase (6PGDH) yield NADPH, a crucial cofactor of the enzyme glutathione reductase (GR) converting glutathione disulfide (GSSG) into its reduced state (GSH). GSH is essential for detoxification processes in and survival of erythrocytes. We explored whether the anti-inflammatory compounds Bay 11–7082, parthenolide and dimethyl fumarate (DMF) were able to completely deplete a common target (GSH), and to impair the function of upstream enzymes of GSH recycling and replenishment. Treatment of erythrocytes with Bay 11–7082, parthenolide or DMF led to concentration-dependent eryptosis resulting from complete depletion of GSH. GSH depletion was due to strong inhibition of G6PDH activity. Bay 11–7082 and DMF, but not parthenolide, were able to inhibit the GR activity. This approach “Inhibitors, Detection of their common target that is completely depleted or inactivated when pharmacologically relevant concentrations of each single inhibitor are applied, Subsequent functional analysis of upstream enzymes for this target” (IDS), can be applied to a broad range of inhibitors and cell types according to the selected target. The specific G6PDH inhibitory effect of these compounds may be exploited for the treatment of human diseases with high NADPH and GSH consumption rates, including malaria, trypanosomiasis, cancer or obesity.

Glucose-6-phosphate dehydrogenase (G6PDH), the rate-limiting enzyme of the oxidative (irreversible) branch of the pentose phosphate pathway (oxPPP), has multiple functions in both pro- and eukaryotic cells. Another NADP^+^-dependent dehydrogenase in glucose-6-phosphate catabolism is 6-phosphogluconate dehydrogenase (6PGDH). In three consecutive enzymatic reactions, G6PDH (reaction 1), followed by 6-phosphogluconolactonase (6PGL, reaction 2) and 6PGDH (reaction 3), glucose-6-phosphate (G6P) is catabolised supplying cells with ribulose-5-phosphate maintaining the antioxidative power by generating 2 NADPH molecules. NADPH is an absolute requirement for reductive metabolism and maintenance of cellular redox homeostasis (Fig. 1).

Long-term inhibition of G6PDH activity and the associated impairment of the NADPH-generating system and glutathione (GSH)-replenishment system significantly increase the vulnerability of the affected cells to apoptosis. Thus, proliferating tumour cells as well as erythrocytes infected with malaria parasites with their high demand for NADPH and GSH can be effectively eliminated by inhibition of G6PDH. Disruption of G6PDH activity has been shown to repress proliferation and simultaneously promote apoptosis in growing tumour cells^1^ and suppress the proliferation of malaria parasites^2^.

Numerous compounds have been used to inhibit the activity of endogenous mammalian G6PDH *in vitro* and/or *in vivo* such as the naturally occurring adrenal steroid dehydroepiandrosterone (DHEA)^3^, catechin gallates, especially epigallocatechin gallate (EGCG)^4^, chelerythrine (initially a PKC inhibitor) and PP2 (Amino-5-(4-chlorophenyl)-7-(t-butyl)pyrazolo[3,4-d]pyrimidine), primarily a Src kinase family inhibitor^5^. Recently, it has been shown that G6PDH from the protozoan parasite Trypanosoma brucei can also be inhibited by DHEA^6^. Our preliminary work with Bay 11–7082, parthenolide or DMF has demonstrated a significant *in vitro* growth inhibitory effect on parasites’ culture of Trypanosoma brucei (own unpublished data). This growth inhibitory effect might also be attributed to G6PDH inhibition.

Many of the hitherto applied inhibitors of G6PDH contain sugar phosphates or various nucleotides competing with the substrate (G6P) or cofactor (NADP^+^), respectively (for review see^7^). In rare cases, a G6PDH inhibition occurs via uncompetitive inhibition, i.e. inhibitor binding to the enzyme-substrate complex. This unusual property has so far been known for DHEA and some closely related steroids (for review see^8^).

G6PDH is an essential enzyme for all cells of the organism limiting its use as preferred drug target. However, there are disease conditions with pathologically enhanced G6PDH activity.

Upregulation of pro-oxidative enzymes NADPH oxidase (NOX) and nitric oxide synthase (NOS), fuelled by G6PDH-derived NADPH, leads to the production of high levels of superoxide anion (O_2_^●−^) in affected subjects with cardiovascular diseases^9^ (for review see^10^), and finally results in premature death. Overexpression of G6PDH renders tumour cells more resistant to cell death^11^. This can be explained by the augmented ribose-5-phosphate production and regeneration of NADPH and GSH pools, and is thus considered as a cancer-promoting process. In addition, the use of G6PDH inhibitors, e.g. DHEA, which disrupt NADPH-dependent lipogenesis is a powerful approach to prevent obesity^12^ and to inhibit spontaneous breast cancer (for review see^8^).

Several groups have already shown inhibition of erythrocyte G6PDH by DHEA *in vivo* and *in vitro*^2^,^13^. The latter work also demonstrated a DHEA-mediated GSH-depletion in human erythrocytes.

Numerous studies have confirmed the central role and importance of G6PDH in cell growth, development, disease progression, senescence, death signalling and its pivotal role as a metabolic nexus in many cellular systems. The present study therefore is based on the assumption that Bay 11–7082, parthenolide and DMF might have the capacity to completely inactivate or deplete one common target, GSH, in pharmacologically relevant concentrations^14^. Targeting of GSH may then influence the cell survival, and in the case of erythrocytes induce eryptosis. For this, we measured GSH levels and performed precise analysis of the relevant enzymes crucial for GSH maintenance. Here, we show that inhibitor treatment of mature human erythrocytes predisposes these cells to eryptosis. These findings can be partially attributed to the inhibitory effect of these substances on G6PDH activity and on the inhibitory effect of two of these substances (Bay 11–7082 and DMF) on glutathione reductase (GR) activity, ultimately resulting in GSH depletion and eryptosis.

## Results

### Induction of eryptosis, cell shrinkage and hemolysis by anti-inflammatory compounds Bay 11–7082, parthenolide and dimethyl fumarate (DMF)

We previously reported on the induction of erythrocyte suicidal death (eryptosis) as a result of GSH depletion after treatment of human erythrocytes with DMF^15^, Bay 11–7082 or parthenolide^16^.

However, the role of the metabolic enzymes responsible for GSH regeneration and their involvement in eryptosis under inhibitor treatment conditions has not been addressed yet. In order to confirm eryptosis induction by the inhibitors and to analyse their death-inducing capacity, erythrocyte populations from the same donors were treated with increasing concentrations of Bay 11–7082, parthenolide or DMF, under otherwise identical conditions. Eryptosis was measured by Annexin V binding to PS translocated to the outer leaflet of the erythrocyte membrane (scrambling). The strongest effect could be observed with Bay 11–7082, followed by parthenolide and DMF (Supplementary Figures 1 and 2A). In parallel, forward scatter (FSC) as an indicator of cell volume changes after treatment was measured. Analogous to the PS exposure data, a marked decrease of the cell volume was observed in erythrocytes treated with Bay 11–7082, and to a lesser extent following treatment with parthenolide and DMF (Supplementary Figures 2B and 3). Thus, inhibitor-treated erythrocytes showed membrane scrambling and cell shrinkage, two main characteristics of eryptosis.

It is known that hemolysis is induced as a result of unspecific drug interaction with erythrocytes^17^. To quantify hemolysis, the hemoglobin concentration was determined in the supernatant of erythrocytes following exposure to different concentrations of Bay 11–7082, parthenolide or DMF. As depicted in Supplementary Figures 2C and 4, Bay 11–7082 (20 μM) triggered significant hemolysis after 24 h, which further increased after 48 h. However, hemolysis after 24 h affected approx. 3% of the total population, and thus was 9-fold lower as compared with the effect of Bay 11–7082 on Annexin V-binding (compare Supplementary Figures 1A and 4A). The same experiments were conducted with parthenolide (Supplementary Figures 1B and 4B) and DMF (Supplementary Figures 1C, 2C and 4C), in which the ratio of hemolysis to eryptosis was even lower as compared with Bay 11–7082. Thus, treatment of erythrocytes with the anti-inflammatory compounds Bay 11–7082, parthenolide or DMF resulted in induction of eryptosis accompanied by a minimal hemolytic effect. In addition, MCV and MCHC data of inhibitor-treated red blood cells (RBCs) demonstrated that the MCV values were decreasing along with a simultaneous increase of the MCHC values. This clearly showed that under these conditions the erythrocyte membrane remained completely intact. Furthermore, it indicated that the inhibitors alter the function of the erythrocytes in a pharmacologically relevant manner (Tables 1 and 2). Finally, MCV and MCHC data of inhibitor-treated RBCs were in accordance with our FACS data (Supplementary Figures 2B and 3).

### Effects of the pharmacological compounds Bay 11–7082, parthenolide and dimethyl fumarate (DMF) on GSH and GSSG levels

The anti-inflammatory substances Bay 11–7082, parthenolide or DMF are capable to directly and irreversibly bind to GSH in a 1:1 ratio thereby completely curbing the intracellular GSH levels^18^,^19^,^20^. Using an indirect enzymatic method of GSH measurement, GSH depletion has already been observed when human erythrocytes were treated with DMF^15^ or Bay 11–7082^16^.

The physiological concentration of GSH is millimolar, whereas that of GSSG is micromolar. Therefore, a painstaking attention to technical details was given for GSH and GSSG analysis, as oxidation of even 1% of a given GSH concentration results in an increase of GSSG of one order of magnitude.

In order to directly measure GSH levels in erythrocytes, we applied the newly developed HPLC-based method of Giustarini *et al*.^21^ (Fig. 2). As shown in Figs 2 and 3a–c, incubation with Bay 11–7082, parthenolide or DMF led to GSH depletion. Human erythrocytes contain 2.5 mM GSH^21^. Therefore, under our experimental conditions (0.6% Hct), the mean concentrations of GSH was 15 μM. However, only Bay 11–7082 was able to totally deplete GSH when used at 20 μM final concentration (i.e. at a higher concentration than GSH), whereas for the other two substances higher concentrations (50 μM and 140 μM, respectively) were needed to almost totally deplete GSH as their common target. As shown in Fig. 3a–c and Table 3, the IC_50_ values of the compounds varied considerably with 7.6 μM for Bay 11–7082, 10 μM for parthenolide and 20 μM for DMF. The affinity of the individual drugs to GSH may differ and thus may result in differing IC_50_ values.

The decrease in GSH and thus the cellular resistance against oxidative stresses observed in inhibitor-treated RBCs was not accompanied by a concomitant increase in GSSG concentration. On the contrary, we could observe a slight decrease also of GSSG, most evident at the highest concentrations of the tested drugs (Table 3). Figure 3d–f illustrates the intraerythrocytic GSH/GSSG ratio following treatment with the compounds. The ratio shows a tendency to decrease by increasing the concentration of the drugs as the consequence of the marked decrease of GSH concentration. Since NADP^+^-dependent redox-relevant enzymes like G6PDH and 6PGDH as well as NADPH-dependent GR function as upstream enzymes of GSH recycling and replenishment, we further investigated the effects of Bay 11–7082, parthenolide and DMF on these enzymes.

### Effects of Bay 11–7082, parthenolide and dimethyl fumarate (DMF) on GSSG level and G6PDH activity

**Figure 1:**
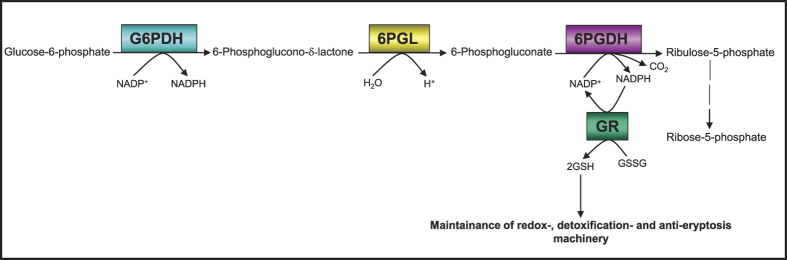
Protection of erythrocytes from oxidative stress-induced eryptosis by G6PDH-GR-Pathway. Providing NADPH by G6PDH ensures GR activity, so maintaining the high intraerytrocytic GSH/GSSG ratio. This protects the cellular thiols as a general requirement for viability. Under these conditions, erythrocytes are protected against oxidative stress-induced eryptosis.

**Figure 2:**
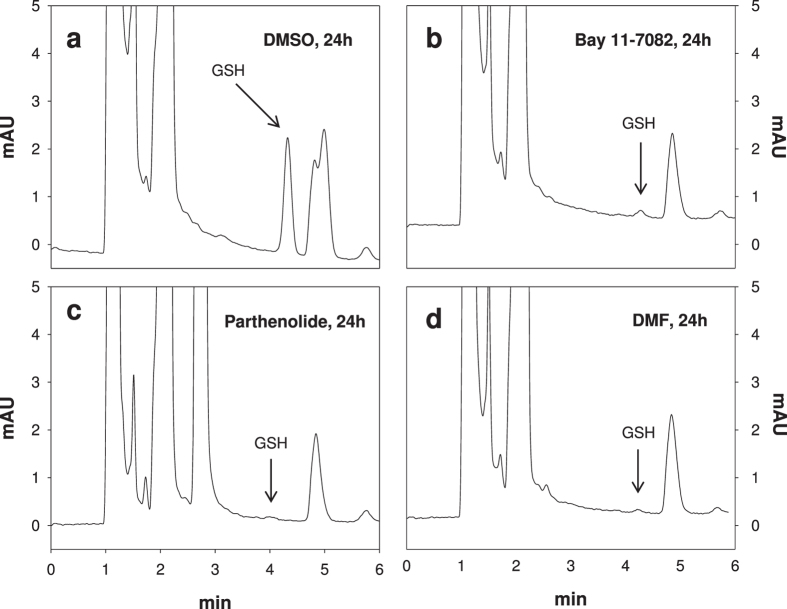
Representative chromatograms obtained for the analysis of GSH in erythrocytes. The GSH-NEM conjugate was analyzed by reverse-phase HPLC with ultraviolet detection at 265 nm (r.t. 4.36 min). GSH depletion in erythrocytes treated with DMSO (0.2% v/v), Bay 11–7082 (20 μM), parthenolide (50 μM) or dimethyl fumarate (DMF: 140 μM) for 24 h are shown.

Several commercially available pro-oxidative substances but also biological/physiological molecules prevalent in cells as for instance hydrogen peroxide (H_2_O_2_) decrease the existing intracellular high GSH/GSSG ratio according to the principle of an inverse correlation in favour of a high GSSG/GSH ratio. This dynamic reciprocal proportionality in mature human erythrocytes between intracellular GSH and GSSG concentrations was not observed, when cells were treated with the anti-inflammatory compounds Bay 11–7082, parthenolide, or DMF. In our study, GSH depletion was not accompanied by an increase of the intracellular GSSG concentration (Table 3) forcing the affected cells to newly produce more GSH as well as to enhanced conversion of the already existing GSSG molecules into GSH, thereby maintaining their redox capacity. For the conversion of GSSG to GSH, cells use the glucose-6-phosphate dehydrogenase-NADPH-glutathione reductase (G6PDH-NADPH-GR) system (Fig. 1).

Newly regenerated GSH molecules are depleted by these compounds via conjugate formation and intracellular concentrations of both GSH and GSSG are diminished depending on the respective concentrations of Bay 11–7082, parthenolide or DMF. As presented in Table 3, at 20 μM Bay 11–7082, 50 μM parthenolide or 140 μM DMF no GSH and only marginal GSSG concentration could be detected.

GSSG as an oxidized form of glutathione and as an electron acceptor for the NADPH- dependent GR effectively stimulates the activity of G6PDH^22^. The latter as the primary enzyme of the oxidative branch of the pentose phosphate pathway (oxPPP) oxidizes its specific substrate, glucose-6-phosphate, by which the first NADPH molecule is produced. The produced NADPH molecule in turn serves as co-factor for GR. As an integral component of the anti-oxidative system, GR converts its physiological substrate GSSG into GSH. Thereby, erythrocytes are protected from oxidative stress-induced eryptosis (Fig. 1).

These basic considerations led us to the assumption that Bay 11–7082, parthenolide or DMF might have a direct influence on the activity of the NADPH-producing enzymes G6PDH, 6PGDH and thus on the activity of the NADPH-dependent flavoprotein, GR. Therefore, we conducted experiments under the same conditions as in Supplementary Figure 1. The results showed that total G6PDH inhibition could be achieved with Bay 11–7082 at 20 μM or with parthenolide at 50 μM, and to a lesser degree (~50% G6PDH inactivation) with DMF at 140 μM (Fig. 4a–c and Table 4).

### Effects of Bay 11–7082, parthenolide and dimethyl fumarate (DMF) on the 6PGDH activity

We further investigated the effect of Bay 11–7082, parthenolide or DMF on the second NADPH generating enzyme, 6PGDH in mature human erythrocytes. We did not observe any inhibitory effect of these compounds on 6PGDH activity at the concentrations used (data not shown), pointing to a specific inhibitory effect on the activity of the first NADPH generating enzyme, i.e. the G6PDH in erythrocytes. The similarities between DHEA as an uncompetitive inhibitor of mammalian and lower eukaryotes G6PDH and the compounds Bay 11–7082, parthenolide and DMF used in our study are intriguing. DHEA exclusively inhibits G6PDH, but not 6PGDH^23^ and caused GSH depletion in mature human erythrocytes^2^.

The idea that Bay 11–7082, parthenolide or DMF might exert hitherto unrecognized regulatory/inhibitory effects on other redox-relevant enzymes, prompted us to investigate a possible inhibitory effect of these compounds on GR activity. Among other functions, G6PDH serves to fuel GR with NADPH. GR, in turn, as the central enzyme of glutathione redox metabolism triggers via its co-factor NADPH the conversion of its physiologic substrate GSSG into GSH.

### Effects of Bay 11–7082, parthenolide and dimethyl fumarate (DMF) on the glutathione reductase (GR) activity

Each monomer of the dimeric mammalian flavoprotein GR is composed by four structural domains: the flavin adenine dinucleotide (FAD) as prosthetic group, NADPH, central and interface domains. The highly reactive site thiols Cys58 and Cys63 constitute a reversible redox system and act as part of the active centre. Thus, these thiols provide GR with a profound reductive capacity to convert its physiological substrate GSSG into its reduced form GSH^24^. Regeneration of GSH in turn protects erythrocytes from oxidative stress-induced eryptosis. Unlike parthenolide, only Bay 11–7082 and DMF were able to inhibit the GR activity (Fig. 4d–f and Table 4). Bay 11–7082 (20 μM) inhibited GR activity by 100% whereas the highest clinically established dose of DMF (140 μM) achieved 60% inhibition of GR activity.

In our experimental set up, however, we observed an interesting phenomenon: Whereas control samples (lysates of the DMSO-treated erythrocytes) and all other inhibitor-treated samples showed an increased GR activity (at least in tendency) after addition of FAD (Fig. 5a–c), the samples previously treated with 20 μM Bay 11–7082, thereby resulting in 100% inhibition of GR, did not demonstrate significantly increased GR activity in the presence of FAD (Fig. 5a). Thus, it seems reasonable that at this pharmacologically established concentration Bay 11–7082 possesses the potential to irreversibly inhibit GR activity (Fig. 5a).

## Discussion

Targeting G6PDH is a pharmacological meaningful approach to treat a variety of pathologic conditions including cancer and parasitic infections. Recently, approximately three million commercially available substances have been scrutinized by various established approaches regarding their putative inhibitory effects on the G6PDH activity. Out of the selected 250 potential candidates studied *in vitro*, only 8 compounds were capable to inhibit more than 50% of the G6PDH activity at concentrations <100 μM^4^. Thus, it has become evident that the search for suitable G6PDH inhibitors remains difficult. This is surprising as the enzyme G6PDH has already been described 80 years ago. With our current study we present three compounds, one of them an already approved drug (DMF), the second tested in clinical phase I trial (parthenolide), all of them inhibiting the G6PDH activity very efficiently: a 100% inhibition with Bay 11–7082 at 20 μM or parthenolide at 50 μM, and to a lesser degree (~50% G6PDH inactivation) with DMF at 140 μM (Fig. 4a–c).

**Figure 3:**
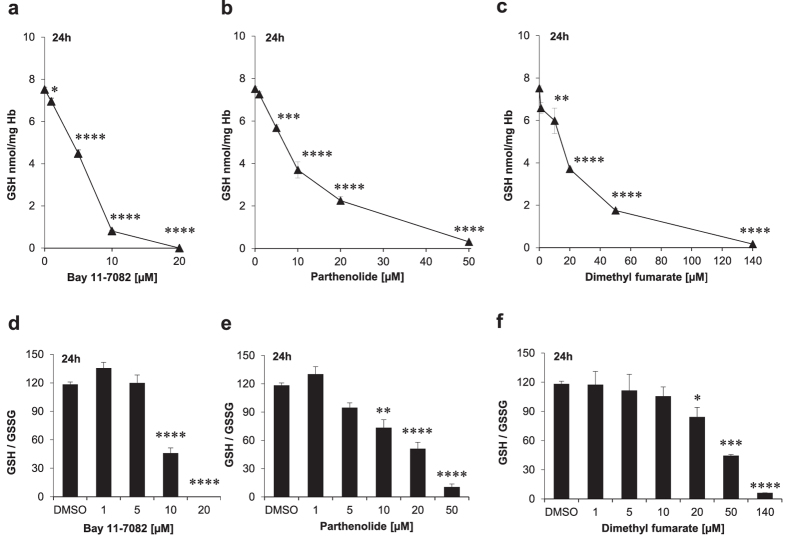
Dose-dependent effect of either Bay 11–7082, parthenolide or dimethyl fumarate (DMF) on glutathione and glutathione disulfide levels of mature erythrocytes. Cells were treated with different concentrations of the compounds in Ringer solution for 24 h. The values shown are the mean ± SEM of three independent experiments with different blood samples.

Fumaderm (a combination drug composed of DMF and monoethyl fumarate (MEF) salts) is registered for treatment of moderate-to-severe psoriasis since 1984 in Germany, while in recent years, Tecfidera was approved in EU and US for treatment of multiple sclerosis. In both inflammatory diseases, the exact mechanisms of action of DMF have not been conclusively deciphered yet, even though the roles of GSH depletion and the switch of different immune cells have been demonstrated^25^. DMF is regarded as a pro-drug with monomethyl fumarate (MMF) and/or the GSH-adducts of DMF and MMF to be the active *in vivo* moieties. Due to high doses that are given orally (120 to 240 mg DMF per tablet) high local concentrations can be assumed after release in the gut lumen. Due to high lipophilicity DMF can penetrate into the mucosa and may affect immune cells and red blood cells in the local vasculature. Unfortunately, there is no published literature about local DMF concentration in the small intestine neither in animals nor in man. Parthenolide, a naturally occurring sesquiterpene lactone exhibits broad-spectrum anti-cancer activities and has already been tested in cancer clinical trials (for review see^26^). Primitive human acute myelogenous leukemia cells show constitutively activated NFκB^27^. These cells with their acquired aberrant GSH metabolism can be effectively eliminated by parthenolide^28^. In this context, Bay 11–7082 with its NFκB inhibitory potential^29^ and its ability to deplete GSH at pharmacologically relevant concentrations (Fig. 3a) might also be taken into account for the treatment of patients with hematologic malignancies and inflammatory diseases. On the other hand, our *in vitro* data showing that all 3 inhibitors also induced programmed erythrocyte death, i.e. eryptosis, argue in favor of a careful clinical use of these substances, as it has been shown earlier that enhanced eryptosis leads to accelerated RBC clearance^30^, whereas inhibition of eryptosis prolongs the half-life of erythrocytes *in vivo*^31^.

**Figure 4:**
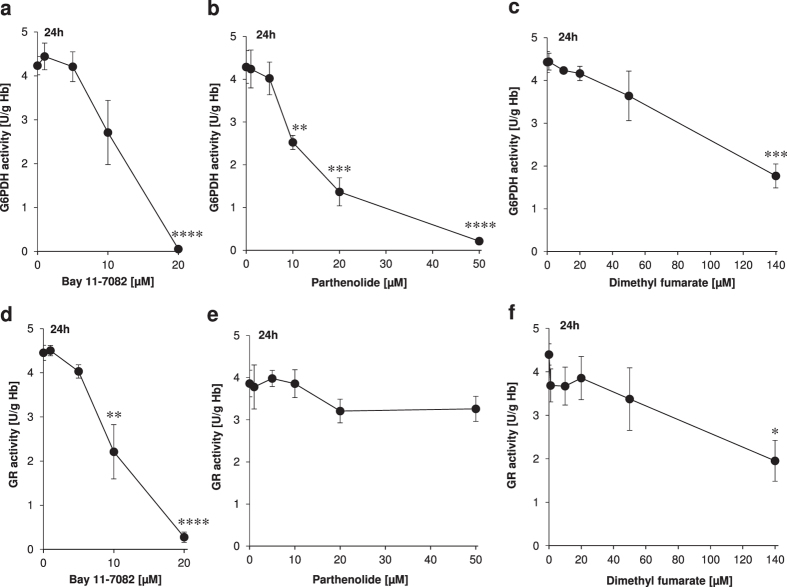
Determination of G6PDH (**a–c**) and GR (**d–f**) activities as functions of Bay 11–7082 (**a,d**), parthenolide (**b,e**) or dimethyl fumarate (DMF) (**c,f**) concentrations. Erythrocytes were treated with Bay 11–7082, parthenolide or DMF for 24 h. DMSO (0.2% v/v)-treated erythrocytes served as negative control. The values shown are the mean ± SEM of three independent experiments each performed with three replicates.

Altered G6PDH activity has multiple pathophysiological consequences. Hyperactivity or constitutive activation of G6PDH, the major producer of extra-mitochondrial NADPH, mediates NADPH-dependent superoxide anion generation (O_2_^●−^)^32^, whereas loss of G6PDH activity is associated with decreased production of O_2_^●− ^33. Cytosolic NADPH as donator of electrons serves to fuel O_2_^●−^ generating enzymes such as NADPH-dependent oxidases (NOXs) which are major sources of O_2_^●−^ production in human myocardium^34^. A continuous increase in intracellular O_2_^●−^ concentrations causes apoptosis of myocardial cells (for review see^35^). Moreover, the generated O_2_^●−^ is metabolised to other reactive oxygen species (i.e. hydrogen peroxide) by superoxide dismutase (for review see^14^,^35^) regarded responsible for premature death of patients with heart failure. Therefore, the pharmacological compounds Bay 11–7082, parthenolide and DMF with their inhibitory effect on the G6PDH activity at non-toxic concentrations (Fig. 4a–c) should not only be considered for the evaluation of the long-term cardiovascular effects of G6PDH inhibition, but also for the treatment of patients suffering from NADPH-superoxide anion associated heart failure.

Hyperactivity or constitutive activation of G6PDH promotes tumour growth. Administration of DHEA prevents tumour development and proliferation^36^. Several *in vitro* studies have already shown the anti-cancer efficacy of Bay 11–7082^ ^37, parthenolide^38^ and DMF^39^. Obviously, a connection of the anti-cancer potential of these compounds and their inhibitory effect on G6PDH and GR activities has been neglected, despite the fact that oxPPP plays a crucial role in the production of NADPH and ribulose-5-phophate, and causes an NADPH-dependent GR activity (Fig. 1). These factors are known not only to be involved in the growth of healthy cells but also in the unrestrained proliferation of growing cancer cells. Moreover, in 50% of all hitherto known human tumours and tumour cell lines, the tumour suppressor protein p53 is fully mutated/inactivated (for review see^40^). It is known that wild type p53 inhibits G6PDH activity directly^41^. p53 mutation/inactivation and the connected hyperactivation of G6PDH might be targeted by the administration of Bay 11–7082, parthenolide or DMF.

The inhibitory effects of these compounds are not restricted to the targets described above, but they may exhibit multifunctional properties, among them the inhibition of the NFκB signalling pathway involved in several inflammatory and survival processes^29^,^42^,^43^. We previously reported about the existence of NFκBs and their upstream kinases in mature human erythrocytes^16^. Our *ex vivo* preliminary work is pointing to a possible anti-apoptotic function of NFκB in erythrocytes (own unpublished data).

At this moment, we are not able to explain the underlying mechanisms how Bay 11–7082, parthenolide or DMF inhibit the activity of the lipogenic enzyme G6PDH. One explanation might be that these compounds influence the upstream regulators of G6PDH phosphorylation and/or dephosphorylation. Numerous studies have already shown the stimulatory or inhibitory effects of phosphorylation on G6PDH activity^44^,^45^.

**Figure 5:**
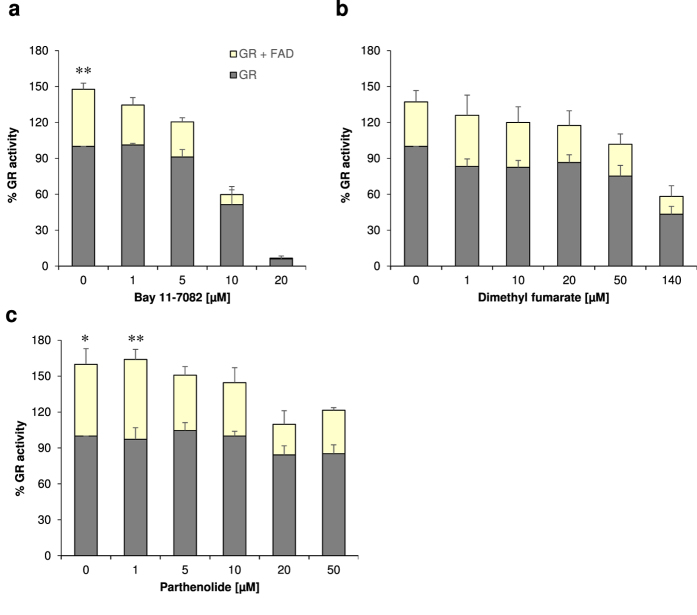
Irreversible inhibition of GR activity with 20 μM Bay 11–7082. Exogenous FAD was added to control samples (lysate of the DMSO-treated erythrocytes) and to identical samples previously treated with Bay 11–7082, parthenolide or dimethyl fumarate (DMF) for 24 h. Differences of identical samples in presence or absence of FAD were analysed by one-way ANOVA comparison and Sidak’s post test.

Human erythrocytes possess a highly active GSH synthesis machinery. Apart from liver^46^, human erythrocytes deliver a considerable amount of GSH molecules into the blood^47^, thus replenishing the GSH pool of the organism. Unlike parthenolide, Bay 11–7082 and DMF were able to inhibit the GR activity (Fig. 4d–f as well as Fig. 5a–c). Inhibition of GR activity drops the intracellular concentration of GSH significantly, thus predisposing erythrocytes to eryptosis.

Mature erythrocytes possess functional eNOS that modulates vascular nitric oxide (NO) signalling *in vivo*^48^. NO is able to convert a large percentage of intracellular GSH [GSH]_i_ to S-nitrosoglutathione (GSNO), thus depleting [GSH]_i_^ ^49. In addition GSNO inhibits the GR activity^50^. Bay 11–7082 51, parthenolide^52^ or DMF^53^ suppress the synthesis of intracellular NO. Furthermore, there is a strong association between G6PDH activation and its influence on NOS activity^54^. G6PDH serves to fuel NOS with NADPH, thus ensuring its full activity to synthesize NO and L-citrulline from the amino acid L-arginine (for review see^10^).

Bay 11–7082-, parthenolide- or DMF-mediated G6PDH inhibition (Fig. 4a–c) may cause a significant reduction of NO production in mature human erythrocytes. This fact together with the complete GSH depletion caused by these compounds (Fig. 3a–c) makes a GSNO-induced GR inhibition very unlikely, at least in our experimental set up.

Bay 11–7082, parthenolide and DMF via their inhibitory effects on the lipogenic enzyme G6PDH (Fig. 4a–c) and GR activities (Fig. 4d,f) and their potential to deplete GSH (Fig. 3a–c) might cause a moderate and, if necessary, strong systemic decrease in the NADPH and GSH production, depending on the concentration used. The systemic effect of these anti-inflammatory compounds and their clinical relevance is versatile. The following reflections should support this concept: (i) erythrocytes are no longer able to contribute to the extracellular pool of GSH. Thus, their cooperation with liver to maintain interorgan GSH metabolism^47^ is disrupted, (ii) in the context of malaria the G6PDH inhibition has the following effect: the unfettered synthesis rate of the ribose-5-phophate and NADPH in Plasmodium *falciparum*-infected erythrocytes (it is 78 times higher than that in uninfected erythrocytes) can be tremendously diminished thereby leading to early death of the parasites (for review see^55^), (iii) on the same basic principle, the unrestrained synthesis of ribose-5-phosphate and NADPH in growing tumour cells can be efficiently inhibited (for review see^56^), (iv) the GSH- and NFκB-mediated irradiation resistance of growing tumour cells can be curbed significantly (for reviews see^57^,^58^), and (v) a moderate and systemic decrease of GSH might prevent diet-induced obesity as previously demonstrated in mice^59^.

**Figure 6:**
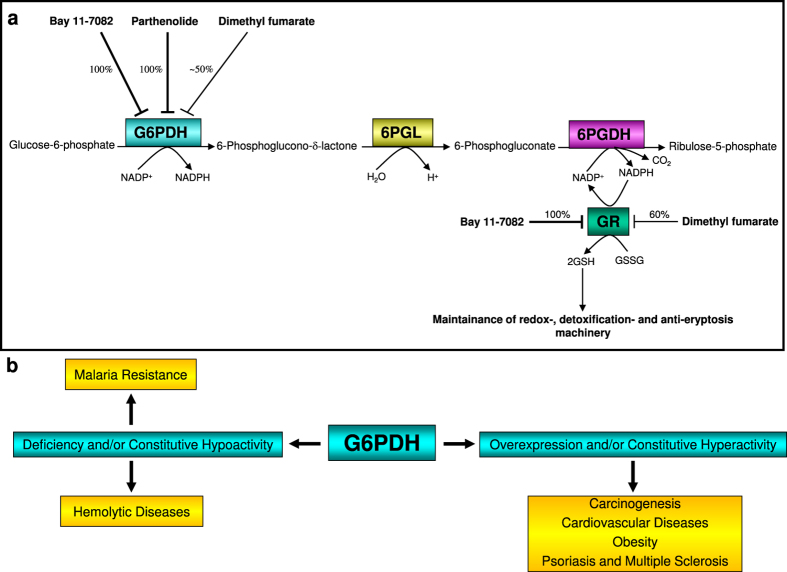
(**a**) Interruption of the oxidative branch of the pentose phosphate pathway (oxPPP) in mature human erythrocytes by the anti-inflammatory compounds Bay 11–7082, parthenolide and dimethyl fumarate (DMF). The susceptibility of erythrocytes to eryptosis mediated by these compounds is partially based on the inhibition of G6PDH and GR activities. (**b**) Use of G6PDH inhibitors Bay 11–7082, parthenolide and/or dimethyl fumarate (DMF) as important metabolic-based therapy for inflammatory diseases.

Our new insights in the mechanism of anti-inflammatory substances may be useful for the treatment of patients with different diseases (obesity, cancer, malaria, psoriasis, multiple sclerosis, etc.), whose progression strongly depends on the bioavailability of NADPH and GSH (Fig. 6a,b).

In summary, our study, which to our knowledge is the first of its kind, provides evidence that the susceptibility of erythrocytes to eryptosis mediated by Bay 11–7082, parthenolide or dimethyl fumarate (DMF), is partially based on the inhibition of G6PDH and GR activities.

## Materials and Methods

### Erythrocytes

Highly purified erythrocyte suspensions from healthy volunteers with white blood cell (WBC) or thrombocyte contaminations below 0.1%^60^ were provided by the blood bank of the University of Tübingen. Aliquots of the individual erythrocyte concentrates were either used directly at 0.4% or 0.6% hematocrit (Hct) or stored at 4 °C for up to one week. Thus, the age of the erythrocyte concentrates used in this work ranged from 8 to 15 days. The Committee approving the experiments, in name, is the ethics committee of the University of Tübingen, given report numbers: 343/2008BO2 and 184/2003V. Written informed consent was obtained from all subjects. The experiments were carried out in accordance with the approved guidelines.

### Solutions and chemicals

Experiments analysing the suicidal death of erythrocytes (0.4% Hct), determinations of glutathione content, MCV and MCHC values, as well as measurements of enzyme activities (0.6% Hct), were carried out in Ringer solution in a total volume of 2, 30, 30, or 13 ml, respectively. Ringer solution was composed of (in mM): 125 NaCl, 5 KCl, 1.2 MgSO_4_, 32 N-2-hydroxyethyl-piperazine-N′-2-ethanesulfonic acid (HEPES)/NaOH (pH 7.4), 5 glucose, and 1 CaCl_2_. Annexin-binding buffer contained (in mM): 125 NaCl, 10 HEPES/NaOH (pH 7.4), and 5 CaCl_2_. Where indicated, Bay 11–7082 (1–20 μM), parthenolide (1–50 μM) and dimethyl fumarate (DMF; 1–140 μM) were added. Dimethyl sulfoxide (DMSO)-treated erythrocytes served as solvent controls (0.2% (v/v) DMSO). Bay 11–7082, parthenolide, DMF, N-ethylmaleimide (NEM), glutathione reductase (GR) and glutathione disulfide (GSSG) were purchased from Sigma (Taufkirchen, Germany). Annexin V-FLUOS was purchased from Roche Diagnostics (Mannheim, Germany). 10 mg Bay 11–7082 and 5 mg parthenolide were dissolved in 965 μl and 400 μl DMSO, respectively to achieve a 50 mM stock solution. These stocks were aliquoted and stored at −20 °C for up to 3 months. 70 mM DMF stock solution (100 mg dissolved in 9.9 ml DMSO) was freshly prepared. All required solutions were freshly prepared immediately before use.

### Phosphatidylserine exposure and forward scatter

Erythrocyte concentrates suspended in 2 ml Ringer solution (0.4% Hct) were treated in the absence or presence of Bay 11–7082, parthenolide or DMF for 24 h or 48 h. After incubation, 2·10^6^ RBCs were washed in 500 μl Annexin-binding buffer. RBC pellets were then vortexed gently to get a homogenous cell suspension. To detect phosphatidylserine (PS) on the outer leaflet of the plasma membrane, these cells were subsequently stained with 32 μl Annexin V-FLUOS at a 1:33 dilution and mixed gently on a vortex mixer. After 20 min incubation in the dark at room temperature, 200 μl of Annexin-binding buffer was added to each sample, thoroughly vortexed to achieve single cell suspensions, and analysed by flow cytometry on a FACS-Calibur (Becton Dickinson, Heidelberg, Germany). Cell volume was determined by forward scatter (FSC), and Annexin V-FLUOS binding was measured in the FL1-channel.

### Hemolysis

RBC concentrates (0.4% Hct) were treated with varying concentrations of Bay 11–7082, parthenolide or DMF for 24 h or 48 h, then hemolysis was determined. Briefly, after incubation, 600 μl suspension containing 1.2·10^7^ RBCs were centrifuged for 4 min at 420 g, 4 °C, and the supernatants were harvested. To quantify hemolysis, the hemoglobin concentration of the supernatant was determined photometrically at 405 nm. The absorption of the supernatant of RBCs lysed in distilled water was defined as 100% hemolysis.

### Meausurement of MCV and MCHC values

Erythrocyte concentrates (0.6% Hct), suspended in 30 ml Ringer solution were treated with Bay 11–7082 (20 μM), parthenolide (50 μM) and DMF (140 μM) for 24 h, respectively. DMSO (0.2% (v/v))-treated erythrocytes served as negative controls. After incubation, samples were centrifuged at 210 g for 10 min at room temperature in order to obtain RBC pellets. Ionomycin (0.25 μM)-treated erythrocytes served as positive controls, and were first incubated in Ringer solution in the absence of ionomycin for 21.5 h. Subsequently, the cells were treated with ionomycin for 2.5 h, and the MCV and MCHC values were determined. The MCV and MCHC analysis was carried out with an ADVIA 120 Hematology System Instrument (Siemens, Germany).

### Intracellular GSH and GSSG measurements

Erythrocyte concentrates (0.6% hematocrit), suspended in 30 ml Ringer solution were treated with varying concentrations of Bay 11–7082, parthenolide or DMF for 24 h. DMSO-treated erythrocytes served as negative controls. After incubation, erythrocytes were treated with 0.6 mM (final concentration) NEM prepared as a 310 mM stock in H_2_O. NEM functioned as the best alkylating agent for GSH conjugation to prevent sample manipulation-induced oxidation of GSH. After a 2-min incubation at room temperature, samples were centrifuged at 210 g for 10 min in order to obtain RBC pellets. These were immediately stored at −20 °C until GSH and GSSG analyses were performed. All measurements were carried out within one week after the treatment. GSH and GSSG were measured in the clear supernatant obtained by treatment of 0.1 ml RBCs with 0.12 ml 15% (w/v) trichloroacetic acid. GSH analysis was carried out by high-performance liquid chromatography (HPLC), as recently reported^61^. Briefly, one aliquot (0.05 ml) of supernatant was loaded onto HPLC and the GS-NEM conjugate was revealed by a diode-array detector at 265 nm. GSSG was measured photometrically by the GSH recycling method with slight modifications^21^. For hemoglobin determination, one aliquot of the RBC suspensions (10 μl) was hemolysed by 1:200 dilution with H_2_O^62^. The HPLC analyses were carried out with an Agilent series 1100 instrument (Agilent Technologies, Milan, Italy). The photometric analyses were performed with a Jasco V-530 instrument (Jasco Europe s.r.l. Cremella, Como, Italy).

### G6PDH, 6PGDH and GR measurements

RBC concentrates (0.6% hematocrit), suspended in 13 ml Ringer solution were treated with varying concentrations of Bay 11–7082, parthenolide or DMF for 24 h. DMSO-treated erythrocytes served as negative controls. Determinations of the enzymatic activities of G6PDH, 6PGDH and GR (+/− FAD) were performed in leukocytes- and platelets-free erythrocyte lysates according to standard biochemical procedures^63^. After 10 min incubation (20 min for GR) at 37 °C the reaction was started by adding substrate or co-factor. Changes in absorbance at 340 nm were recorded at 1 min intervals for 20 min (Infinite 200 Nanoquant spectrophotometer; Tecan, Männedorf, Switzerland). Specific activity of all enzymes was expressed as U/g Hb. The combined activity of G6PDH and 6PGDH was determined by estimating the conversion of NADP^+^ to NADPH in the presence of glucose-6-phosphate (600 μM). The activity of 6PGDH alone was then measured by the conversion of NADP^+^ to NADPH in the presence of 6-phosphogluconate (600 μM). Finally, G6PDH activity was calculated by subtracting 6PGDH activity from total dehydrogenase activity.

### Statistical analysis

Data are presented as the mean values ± SEM of at least 3 independent experiments with different blood samples. A total of 18 different blood samples were used in this study. Unless otherwise stated, one-way ANOVA with Dunnet’s post test was used for statistical comparisons of treated samples with controls. Differences of the means were considered to be statistically significant when the calculated p value was less than 0.05 (*P < 0.05, **P < 0.01, ***P < 0.001, ****P < 0.0001).

## Additional Information

**How to cite this article**: Ghashghaeinia, M. *et al*. Pharmacological targeting of glucose-6-phosphate dehydrogenase in human erythrocytes by Bay 11–7082, parthenolide and dimethyl fumarate. *Sci. Rep.* **6**, 28754; doi: 10.1038/srep28754 (2016).

## Supplementary Material

Supplementary Information

## Figures and Tables

**Table 1:** MCV and MCHC of inhibitor-treated erythrocytes.

Test	Compounds (24 h incubation)				2.5 h incubation	Blood Groups
	DMSO 0.2% (v/v)	Bay 11–7082 (20 μM)	Parthenolide (50 μM)	DMF (140 μM)	Ionomycin (0.25 μM)	
MCV	85.9	84.1	84.1	85.6	73.3	A Rhesus +
MCHC	33.1	33.9	33.5	33.3	53	A Rhesus +
MCV	83	78.5	82.2	83.5	72.2	B Rhesus +
MCHC	34	37	34.4	33.5	50.4	B Rhesus +
MCV	87.5	86.3	87	88.2	75.8	O Rhesus +
MCHC	32.4	34.1	33.8	32.9	55.7	O Rhesus +
Hct: 0.6%						
Units for MCV: fl						
Units for MCHC: g/dL						

Mature Human Erythrocytes were treated with DMSO (0.2% (v/v), Bay 11–7082 (20 μM), parthenolide (50 μM) or dimethyl fumarate (DMF: 140 μM). Ionomycin (0.25 μM)-treated erythrocytes served as positive controls, and were first incubated in Ringer solution in the absence of ionomycin for 21.5 h. Subsequently, the cells were treated with ionomycin for 2.5 h.

**Table 2:** GeoMean of inhibitor-treated erythrocytes.

Test	Compounds (24 h incubation)				2.5 h incubation	Blood Groups
	DMSO 0.2% (v/v)	Bay 11–7082 (20 μM)	Parthenolide (50 μM)	DMF (140 μM)	Ionomycin (0.25 μM)	
GeoMean	471.7	422.4	425.24	426.7	209.3	A Rhesus +
GeoMean	446	339.3	418.8	427.3	166.2	B Rhesus +
GeoMean	485	410.7	451.5	448	186.3	O Rhesus +
Hct: 0.6%						

Mature Human Erythrocytes were treated with DMSO (0.2% (v/v), Bay 11–7082 (20 μM), parthenolide (50 μM) or dimethyl fumarate (DMF: 140 μM). Ionomycin (0.25 μM)-treated erythrocytes served as positive controls, and were first incubated in Ringer solution in the absence of ionomycin for 21.5 h. Subsequently, the cells were treated with ionomycin for 2.5 h.

**Table 3:** Depletory effect of the pharmacological compounds Bay 11–7082, parthenolide and dimethyl fumarate (DMF) on the intracellular GSH and GSSG concentrations in mature human erythrocytes.

	DMSO 0.2% (v/v)	Bay 11–7082 (20 μM)	Parthenolide (50 μM)	DMF (140 μM)
GSH [nmol/mg Hb]	7.52	0.00	0.30	0.17
GSSG [nmol/mg Hb]	0.06	0.03	0.03	0.03

Hb: haemoglobin.

**Table 4:** Targeting of the glucose-6-phosphate dehydrogenase (G6PDH) and glutathione reductase (GR) in mature human erythrocytes by Bay 11–7082, parthenolide and dimethyl fumarate (DMF).

	Target	EC_50_	EC_100_
Bay 11–7082 (20 μM)	G6PDH	10	20
Parthenolide (50 μM)	G6PDH	12	50
DMF (140 μM)	G6PDH	140	–
Bay 11–7082 (20 μM)	GR	10	20
DMF (140 μM)	GR	140	–

The different percentages of inhibition of the G6PDH and GR activities achieved by each inhibitor are indicated as EC_50_ and/or EC_100_ values.
