# Supplementary material for: Pharmacological targeting of glucose-6-phosphate dehydrogenase in human erythrocytes by Bay 11–7082, parthenolide and dimethyl fumarate
Source: Sci Rep. 2016 Jun 29;6:28754. doi: 10.1038/srep28754 (PMC4926109; doi:10.1038/srep28754)
Supplement: Supplementary Information [file srep28754-s1.pdf]

Pharmacological targeting of glucose-6-phosphate dehydrogenase in human erythrocytes by Bay 11-7082, parthenolide and dimethyl fumarate

Mehrdad Ghashghaeinia[1*], Daniela Giustarini[2], Pavla Koralkova[3], Martin Köberle[4], Kousi Alzoubi[5], Rosi Bissinger[5], Zohreh Hosseinzadeh[6], Peter Dreischer[7], Ingolf Bernhardt[8], Florian Lang[5], Mahmoud Toulany[9], Thomas Wieder[10], Renata Mojzikova[3], Ranieri Rossi[2], Ulrich Mrowietz[1]

[1]Psoriasis-Center, Department of Dermatology, University Medical Center Schleswig-Holstein, Campus Kiel, Schittenhelmstr. 7, Kiel, 24105, Germany; [2]Department of Life Sciences, Laboratory of Pharmacology and Toxicology, University of Siena, Via A Moro 2, 53100, Siena, Italy; [3]Department of Biology, Faculty of Medicine and Dentistry Palacky University, Hnevotinska 3, 77515 Olomouc, Czech Republic; [4]Department of Dermatology and Allergy, Biedersteinerstr. 29, Technische Universität München, 80802 München, Germany; [5]Department of Cardiology, Vascular Medicine and Physiology, University of Tübingen, Gmelinstr. 5, 72076, Tübingen, Germany; [6]Centre for Ophthalmology, Institute for Ophthalmic Research, Eberhard-Karls-University, Tübingen, Germany; [7]Institute of Physiology II, Eberhard Karls University of Tübingen, Keplerstr. 15, 72074 Tübingen, Germany; [8]Saarland University, Campus A2.4, 66123 Saarbrücken, Germany; [9]Division of Radiobiology and Molecular Environmental Research, Department of Radiation Oncology, Eberhard Karls University Tübingen, Roentgenweg 11, 72076 Tübingen, Germany, [10]Department of Dermatology; Eberhard Karls University; Tübingen, Germany

* Corresponding author

Supplementary Information File

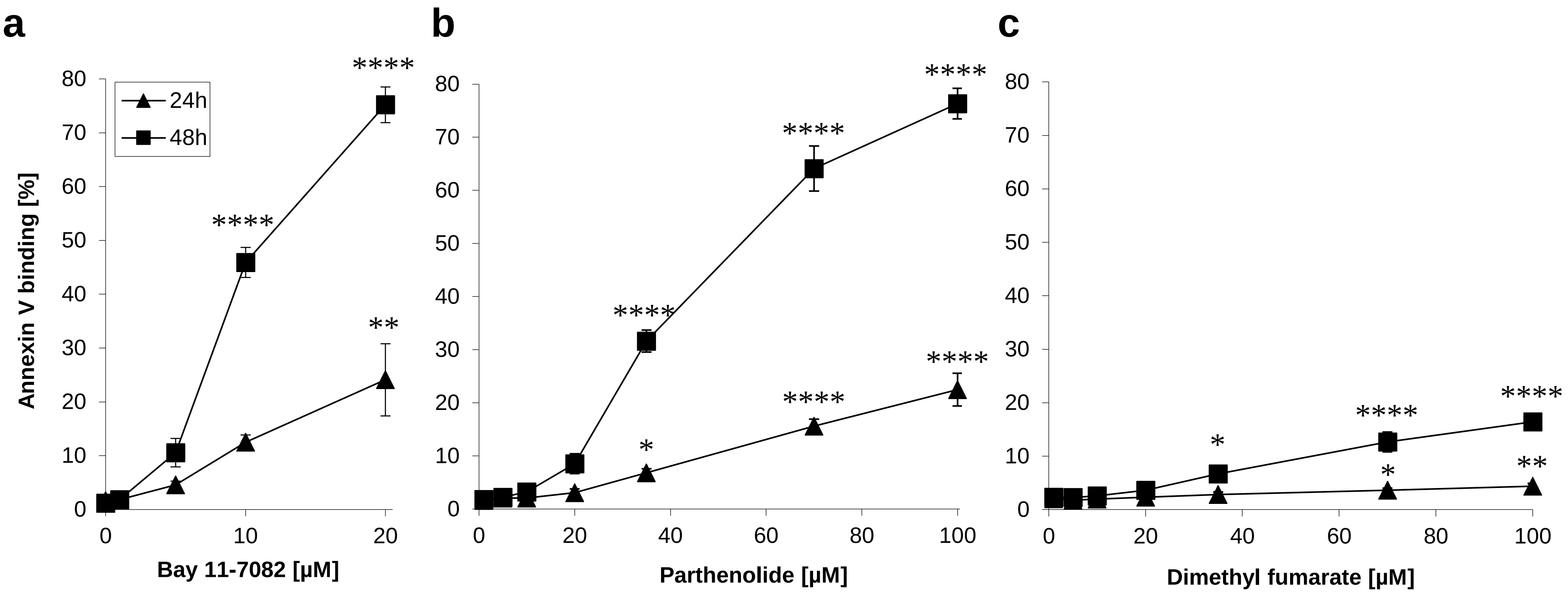

Supplementary Figure 1

Effects of Bay 11-7082 (**A**), parthenolide (**B**) and dimethyl fumarate (DMF) (**C**) on eryptosis. Erythrocytes were exposed either to Bay 11-7082 (1-20 µM), parthenolide (1-100 µM) or DMF (1-100 µM) in Ringer solution for 24 h and 48 h. DMSO (0.2% v/v)-treated erythrocytes served as negative control. The values shown are the mean ± SEM of three independent experiments each performed with three replicates.

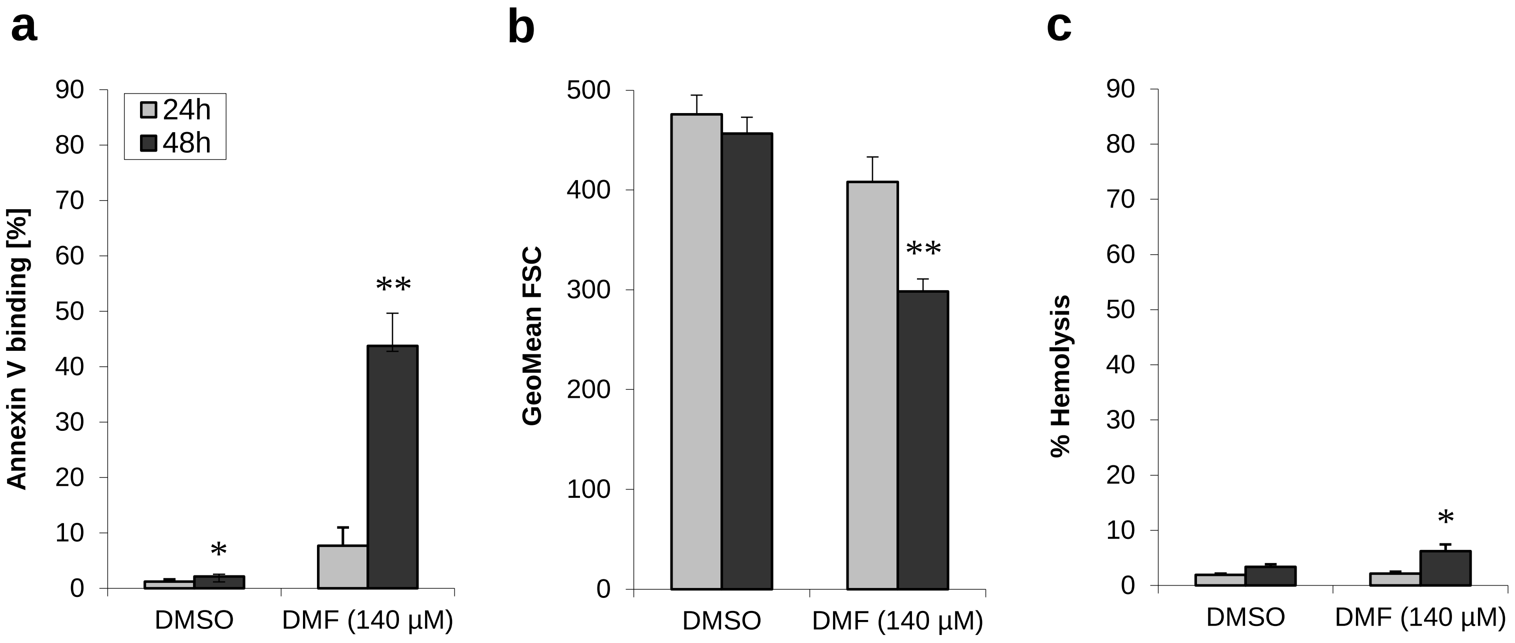

Supplementary Figure 2

Effects of dimethyl fumarate (DMF) (**A**) on eryptosis, (**B**) cell shrinkage and (**C**) hemolysis. Erythrocytes were exposed to DMF (140 µM) in Ringer solution for 24 h and 48 h. DMSO (0.2% v/v)-treated erythrocytes served as negative control. Annexin V assay and hemolysis were performed as outlined in material and methods. The values shown are the mean ± SEM of three independent experiments each performed with three replicates.

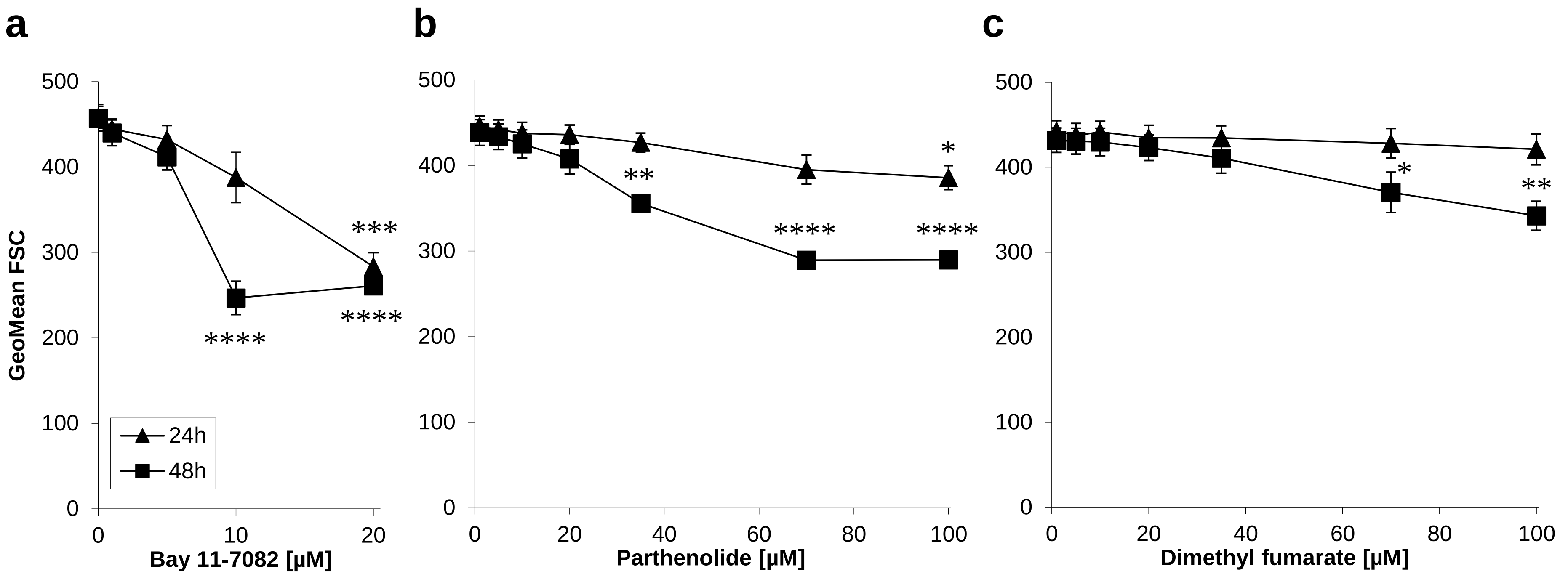

Supplementary Figure 3

Effects of Bay 11-7082 (**A**), parthenolide (**B**) and dimethyl fumarate (DMF) (**C**) on cell shrinkage. Erythrocytes were exposed either to Bay 11-7082 (1-20 µM), parthenolide (1-100 µM) or DMF (1-100 µM) in Ringer solution for 24 h and 48 h. DMSO (0.2% v/v)-treated erythrocytes served as negative control. The values shown are the mean ± SEM of three independent experiments each performed with three replicates.

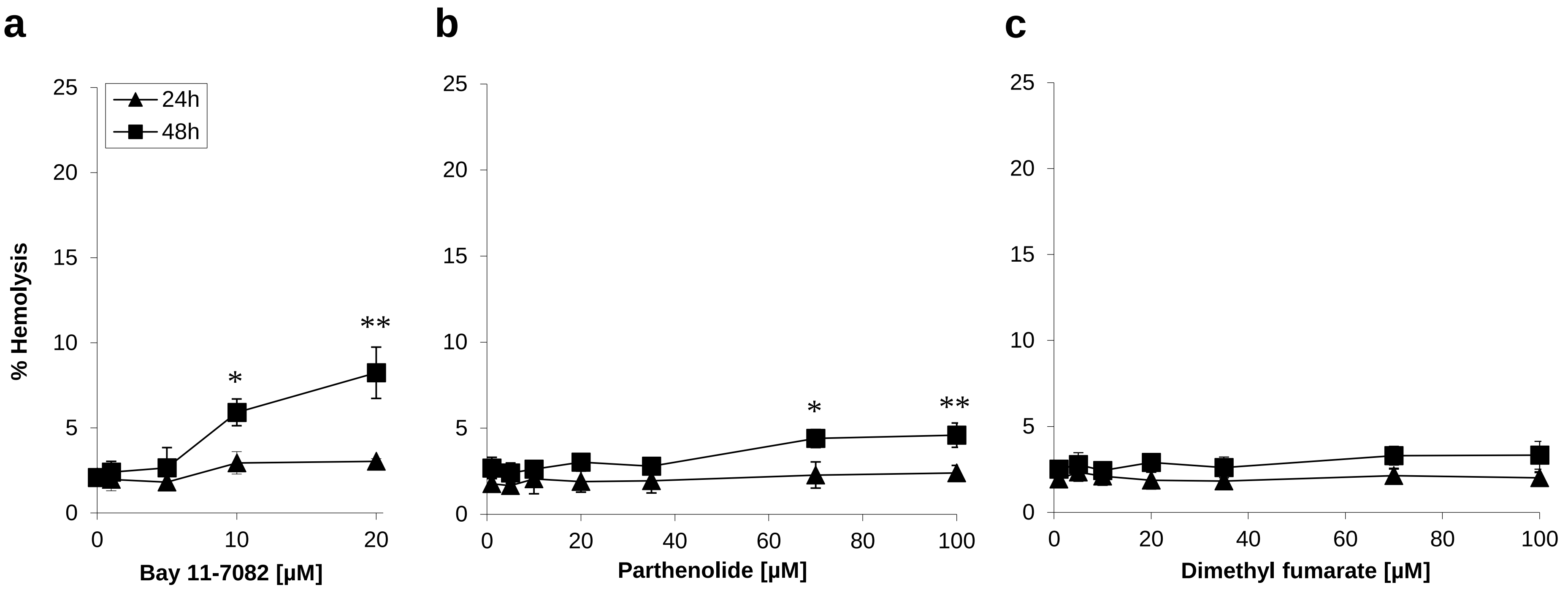

Supplementary Figure 4

Impact of Bay 11-7082 (A), parthenolide (B) and dimethyl fumarate (DMF) (C) on hemolysis. Hemolysis intensity was measured as the increase in turbidity of the erythrocytes´ supernatants, harvested from the corresponding erythrocytes´ suspensions already treated with DMSO or with increasing concentrations of the inhibitors. The values shown are the mean ± SEM of three independent experiments each performed with three replicates.
